# Identification and Selection of Reference Genes for Quantitative Transcript Analysis in *Corydalis yanhusuo*

**DOI:** 10.3390/genes11020130

**Published:** 2020-01-27

**Authors:** Zhenzhen Bao, Kaidi Zhang, Hanfeng Lin, Changjian Li, Xiurong Zhao, Jie Wu, Sihui Nian

**Affiliations:** 1 School of pharmacy, Jiangsu Health Vocational College, Nanjing 211800, China; cicistaying@163.com; 2 School of Life Science and Technology, China Pharmaceutical University, Nanjing 210009, China; 3 School of Pharmacy, China Pharmaceutical University, Nanjing 210009, China; 4 Institute of Modern Chinese Medicine, School of Pharmacy, Wannan Medical College, Wuhu 241002, China

**Keywords:** qPCR, *Corydalis yanhusuo*, reference genes, geNorm, NormFinder, BestKeeper

## Abstract

*Corydalis yanhusuo* is a medicinal plant frequently used in traditional Chinese medicine, which has effective medical effects in many aspects. Real-time polymerase chain reaction (RT-PCR) has been one of the most widely used methods in biosynthesis research due to its high sensitivity and quantitative properties in gene expression analysis. To obtain accurate normalization, reference genes are often selected in advance; however, no reference genes are available in *C. yanhusuo*. Herein, 12 reference gene candidates, named cyclophilin 2 (*CYP2*), elongation factor 1-α (*EF1-α*), protein phosphatase 2 (*PP2A*), SAND protein family (*SAND*), polypyrimidine tract-binding protein (*PTBP*), TIP41-like protein (*TIP41*), lyceraldehyde-3-phosphate hydrogenase (*GAPDH*), ubiquitin-conjugating enzyme 9 (*UBC9*), cyclophilin 1 (*CYP1*), tubulin beta (*TUBA*), thioredoxin (*YLS8*), and polyubiquitin 10 (*UBQ10*), were selected for stability analysis. After being treated with hormone, UV, salt, metal, oxidative, drought, cold (4 °C), and hot stresses (40 °C), the qRT-PCR data of the selected genes was analyzed with NormFinder, geNorm, and BestKeeper. The result indicated that *GAPDH, SNAD*, and *PP2A* were the top three most stable reference genes under most treatments. This study selected and validated reliable reference genes in *C. yanhusuo* under various environmental conditions, which can provide great help for future research on gene expression normalization in *C. yanhusuo*.

## 1. Introduction

*Corydalis yanhusuo* is a plant from the Papaveraceae family and is widely used to treat drug addiction and as analgesics in China [1]. Abundant pharmacological effects on humans have been identified in the extracts of *C. yanhusuo* (tuber), such as pain relief, anti-tumor effects, and promotion of blood circulation [2,3]. Benzylisoquinoline alkaloids are the main biological components produced by *C. yanhusuo*, and previous studies have found that dehydrocorybulbine, which is found in the extract of *C. yanhusuo*, is a kind of alkaloid that can exhibit antagonistic activity by acting on dopamine receptors [4,5]. Moreover, further research suggests that another active ingredient, tetrahydropalmatine, has a significant effect on protecting against acute global cerebral ischemia-reperfusion injury [6]. However, few studies have focused on the gene expression of *C. yanhusuo*. Some pivotal genes involved in the biosynthetic pathways of important components are not yet clear. For example, various medical functions have been proven by previous research for D-glaucine, but the genes involved in this biosynthesis pathway are still undefined [7]. Also, since some genes are still unknown, studies of gene expression patterns and transcription regulation under a variety of exogenous regulators will be stagnant and blocked [8]. For these reasons, detailed studies are needed to carry out on the genetic discovery and functional verification of *C. yanhusuo*.

High-throughput, or next-generation sequencing (NGS), is a kind of biological technology that has been used for genome sequencing, transcriptome analysis (RNA-seq), DNA–protein interactions (ChIP sequencing), epigenome characterization, and genome re-sequencing [9]. Due to its characteristics of being rapid and low cost in the analysis of the genome and transcriptome of biological organisms, it has become a frequently used biological technology [10]. Quantitative real-time polymerase chain reaction (qRT-PCR) technology is a derivative method, which can monitor the amplification process of target DNA during PCR and it can also be quantitative. It provides an effective and rapid way to quantify the expression level of target genes in various samples by detecting the expression levels of specific reference genes. Relative quantification is the most commonly used quantitative strategy, in which data is standardized by internal control genes. Otherwise, the results would be unavoidably influenced by both internal and external factors, like DNA contamination, RNA purity, complementary DNA (cDNA) quality, primer design, and PCR efficiency [11,12]. The reference gene or housekeeping gene (HKG) is a kind of constitutive internal control gene that can express steadily in different tissues, various samples, and under different environmental pressures, providing a constant reference during quantification of targeted genes [13]. Therefore, to obtain effective results and interpretation from data, selecting an ideal reference gene is vital [14]. The commonly used reference genes in plants are *GAPDH*, actin (*ACT*), 18S rRNA, *CYP*, and alpha-tubulin (*α-TUB*) [15,16,17,18,19]. Nevertheless, several studies demonstrated that the expression levels of traditional reference genes varied widely in different circumstances, which means that they may become unsuitable for data normalization [20,21]. To solve the problem of unsteadiness of internal reference genes in different organisms or under different treatments, some new reference genes, which are expressed at an unchanging level, have been selected for qRT-PCR normalization. There are also studies that have tried to determine which gene is the most stable in a specific organism, such as *Arabidopsis pumila*, *Cichorium intybus*, *Caenorhabditis elegans*, sugarcane, and *Cyprinus carpio* [22,23,24,25,26]. However, there are no systematic studies aimed at the selection of reference genes in *C. yanhusuo* under multifarious abiotic treatments. Therefore, our study reports the first guidance on choosing suitable reference genes in *C. yanhusuo* under a series of environmental stresses for further studies in quantifying genes of interest.

For the aim of obtaining a more circumstantial analysis of the expression stability of reference genes, *C. yanhusuo* plants were pretreated under several external abiotic treatments, including methyl jasmonate (MeJA), UV radiation, NaCl, CuSO_4_, H_2_O_2_, cold, PEG, and H_2_O. The cycle threshold (Ct) values were processed and analyzed by three statistic algorithms: geNorm, NormFinder, and BestKeeper [11,16,27]. These types of software have been widely used to select stable reference genes since 2002 [11,16,27]. geNorm is a software that can process and analyze data. It can evaluate the reliability of reference gene candidates using the expression stability value (M) as a parameter [26]. For each control gene, the pairwise variations of candidates were defined as the logarithmically transformed expression ratio of standard deviation and gene-stability measure. A gene with a lower M value means that it has a more stable expression level. NormFinder is an algorithm that has a similar data processing mode as geNorm. Similar to geNorm analysis, a lower expression stability value (M) refers to higher stability and a higher M value represents lower stability [16]. BestKeeper (https://www.heartcure.com.au/for-researchers/) can calculate the Ct values to analyze variabilities in each candidate reference gene [27]. RefFinder is a web-based comprehensive tool (https://www.heartcure.com.au/for-researchers/). It integrates the currently available major computational programs (geNorm, Normfinder, BestKeeper) to compare and rank the tested candidate reference genes. To confirm the stability of selected genes, RNA-seq data based on gene expression profiling was used to compare the sorting results. Meanwhile, the 1-aminocyclopropane-1-carboxylate oxidase (*ACO*) gene was used as a standard to validate the stability of selected genes since the expression level of *ACO* is generally considered to be steady. In general, the result of the study would play a significant role in the study of *C. yanhusuo*, especially for the studies of genes involved in biosynthesis.

## 2. Materials and Methods

### 2.1. Plants and Growth Environments

One-year-old *C. yanhusuo* were transplanted from the botanical garden (Medicinal Botanical Garden of China Pharmaceutical University) to pots (diameter 15 cm) containing a mixture of perlite, vermiculite, and peat moss at a ratio of 1:1:1 in the laboratory and cultured for seven days under the same conditions. The plants were kept at 20 °C, with a day length of 12 h (H), and were watered regularly. The relative humidity was maintained between 40% and 70%. For hormone treatment, MeJA (purchased from Aladdin, Shanghai, China), was dissolved in 95% ethanol to make a stock solution; then, it was diluted into 25 mM with ddH_2_O for use. Plants were subjected to 200 mL MeJA for 6 h before harvest. Salt stress treatment was applied by using 200 mL of 500 mM NaCl for seven days. Oxidative stress was carried out by exposing the leaves to 200 mL of 50 mM H_2_O_2_ for 24 h. To apply cold and hot stress treatments, plants were placed in an illuminating incubator at 40 and 4 °C for 48 h, respectively. Metal treatment was carried out by using 200 mL of 200 mM CuSO_4_ solution for 24 h. For drought treatment, 200 mL of 20% PEG 4000 were used per day to water the plant for seven days. For the ultraviolet rays (UV) treatment, a monochromatic lamp (312 nm) was used to irradiate plants with a set distance of 15 cm, and the plants were rotated every 2 h to minimize positional effects. Control groups were treated with distilled water. All solutions were poured into the soil directly, and all experimental groups under different treatments contained three biological replicates and three technical repeats for expression analysis. The harvested sample tender leaves were frozen in liquid nitrogen prior to the degradation of mRNA and then stored at −80 °C.

### 2.2. RNA Isolation and Complementary DNA Synthesis

An EASY spin Universal Plant RNA Kit (Aidlab, Beijing, China) was used to extract RNA by using about 100 mg of frozen sample. Then, the quality and purity of the total RNA samples were detected by the NanoDrop spectrophotometer 2000 (Thermo Fisher Scientific, Waltham, MA, USA)—only RNAs with optical density OD_260/280_ ratios ranging from 1.8–2.1 and an OD_260/230_ ratio between 1.6 and 2.2 were used for further analysis. To eliminate the influence of DNA contaminants, RNAase-free DNAase I (Takara Biotechnology, Dalian, China) was used to pretreat RNA samples before being used in reverse transcription. By following the guidance (HiScript Q RT SuperMix for qPCR, Vazyme, China), 1 μg of template RNA was used for cDNA synthesis in a 20-mL admixture and then diluted five times for qRT-PCR analyses.

### 2.3. Selection of Candidate Reference Genes and Primers Design

A total of 12 genes were chosen as candidate genes to determine the most suitable reference gene in *C. yanhusuo* under multiple environmental pressures. Half of the candidates were selected according to previous research (*CYP2*, *PP2A*, *TIP41*, *UBQ10*, *CYP1*, *TUBA*, *GAPDH*) [28,29,30] and another half (*EF-1α*, *PTBP*, *SAND*, *UBC9*, *YLS8*) were selected by comparison with the TAIR database (http://www.arabidopsis.org). To select and screen the potential unigenes, the internal program in Bioedit Sequence Alignment Editor was used in the local BLAST; https://blast.ncbi.nlm.nih.gov/). The information of the *C. yanhusuo* sequence has been uploaded to the BioProject database of National Center for Biotechnology Information (Access Number is PRJNA539894, all sequence information of *C. yanhusuo* is shown in Table S1). By using the TAIR database, potential homologs of 12 genes were selected, and a high bit score with a low *E*-value was the standard to choose genes. Primers had to come across the exon–intron boundaries, and the AlignX program in vector NTI advance 11.5 was used to perform the exon analysis to avoid the DNA pollutant. All primers were designed based on the following criteria: The length of amplification was between 100 and 150 bp, the GC content range was from 40–60%, the length of primer ranged from 17 to 25 bp, and the differences in the melting temperature (T_m_) between the forward primer and reverse primer were less than 1 °C. The information of the primer pairs involved in this research is presented in Table 1.

### 2.4. Quantitative Real-Time Polymerase Chain Reaction (qRT-PCR) Analysis

The reaction system contained 10 μL of Vazyme qPCR SYBR Green Master Mix (No Rox, Vazyme, Nanjing, China), 2 μL of 5 times diluted cDNA, and sterile ultrapure water added to 20 μL. The reaction was carried out under the following cycle conditions: Five minutes in 95 °C for 1 cycle, and then 10 s in 95 °C, 20 s in 55–60 °C, and 20 s in 72 °C for 40 cycles. The qRT-PCR reactions were performed three times for analytical replicates. Each stress treatment had three biological replicates and Table S2 shows the raw Ct values of all candidates’ reference genes. Then, melting curve analysis was used to detect the specificity of each primer pair because only results with high reaction specificity can be used for quantitative results analysis. The LC480 Conversion and LinRegPCR programs [31,32,33,34] were used to obtain quantitative PCR amplification efficiencies. Fluorescence intensity and cycle values were compared and analyzed by the LC480 conversion and LinRegPCR programs to be able to quantify the relationship between them.

### 2.5. Statistical Analysis of Gene Expression Stability 

In order to visually exhibit the stability of each candidate gene under different experimental conditions, geNorm, NormFinder, and BestKeeper were used to process the raw Ct values obtained by qRT-PCR. Data for the geNorm and NormFinder algorithms had to be processed by the formula: 2^−ΔCt^ (ΔCt = each corresponding Ct value − the minimum Ct value). Then, by importing 2^−ΔCt^ values into the programs, the stability parameters of each gene could be obtained. In geNorm analysis, the stability value (M) could be generated by comparing the pairwise variation (V) between different candidate genes. The threshold was often used as a parameter to evaluate the stability of expression of candidate genes and higher M values refer to worse stability. Pairwise variation (V_n_/V_n+1_) analysis was performed as a desired value that suggests the number of candidate genes needed for accurate normalization [11]. When this value was less than 0.15, it indicated that the number of internal reference gene combinations in this group can maintain the accurate normalization of the data to some extent. NormFinder is rooted in a mathematical model that assesses the reliability of candidate genes and estimates variations in both the intra-group and inter-group [30]. In geNorm, the gene with higher expression stability usually exhibits lower expression stability values (M), which is the same as NormFinder. BestKeeper uses a different mechanism to choose the most stable genes; CV ± SD (coefficient of variation ± standard deviation) is used as the parameter, and genes with low parameters are considered to have high stability. Through the comparative analysis of three kinds of analytical software, it is intuitive to select the internal reference genes more accurately under different external environments.

### 2.6. Comprehensive Analysis and Validation of Selected Reference Genes

To validate the outcomes of the NormFinder, geNorm, and BestKeeper, a comprehensive ranking platform RefFinder was used to identify the most reliable gene under various environmental conditions. For the aim of verifying the reference genes identified in this study, primers were designed according to the RNA-seq of *ACO*, and qRT-PCR technology was performed to quantify the relative abundance of *ACO* (it can be extracted from PRJNA539894) under MeJA treatments for confirmation of the reliability of this study. qRT-PCR data were obtained by performing three biological replicates. The obtained data were processed based on the 2^−△△Ct^ method to transfer to the relative expression level [28].

## 3. Results

### 3.1. Evaluation of Amplification Specificity and PCR Efficiency in C. yanhusuo 

In order to survey the reference gene of *C. yanhusuo*, 12 candidate genes (*CYP2*, *EF1-α*, *PP2A*, *PTBP*, *SAND*, *TIP41*, *UBC9*, *UBQ10*, *CYP1*, *TUBA*, *YLS8*, and *GAPDH*) were screened according to previous studies [29,30,35] and the TAIR database. *Arabidopsis* homolog locus, PCR efficiency, gene symbol, amplicon length, and correlation coefficients (R^2^) are shown in Table 1. In addition, the melting curve analysis confirmed the specificity of genes since only one single peak was formed (Figure S1). By following the LinRegPCR program [31,32,33,34], the average amplification efficiency (E) of primers was from 1.598 to 1.860. The regression coefficient used the same slope with the standard curve, and R^2^ (correlation coefficients) ranged from 0.676 to 1.000 (Table 1). Of them, TUBA has the lower primer efficiency. Considering TUBA belongs to the tubulin protein superfamily, which is different from other protein types, and TUBA shows high stability in some species, we also used it as the candidate reference gene in the subsequent experiments.

### 3.2. Expression Profiles of Reference Genes

**Figure 1:**
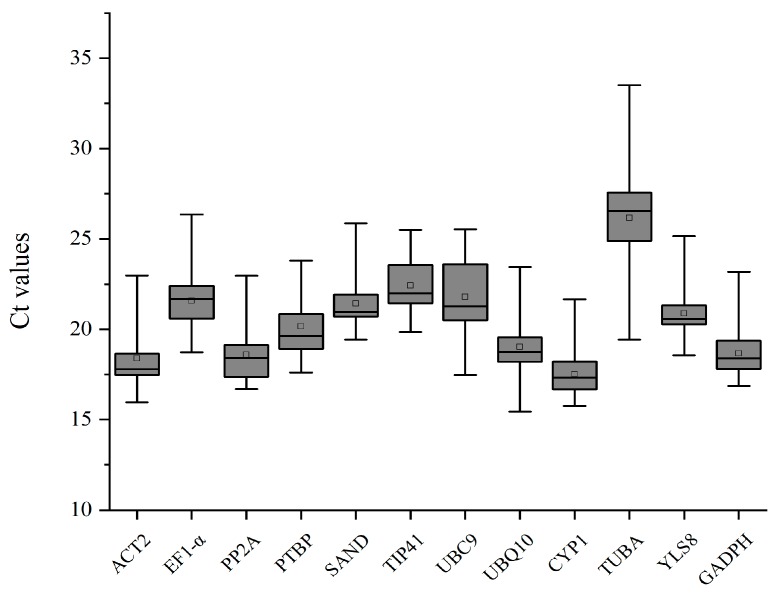
The raw cycle threshold (Ct) values of all candidate reference genes. The boxes show two interquartile values. Whisker caps denote the maximum and minimum Ct values. Medians and means are indicated by the lines and squares, respectively.

The Ct values indicate the cycles required to reach the threshold; genes with lower Ct values represent higher expression levels. The mean Ct values of candidates ranged from 17.51 to 26.16 and most of them were distributed from 18 to 22. *CYP2*, *PP2A*, and *GAPDH* showed the greatest potential as they had the lowest Ct values (mean ± SD) of 18.38 ± 1.71, 18.58 ± 1.62, and 18.66 ± 1.23, respectively. *TUBA* was the least abundant candidate because it had the largest Ct value (26.16 ± 2.44) (Figure 1 and Table S2). In addition, *TUBA* exhibited a high variability since the SD can reflect the dispersion degree of a data set, and *TUBA* possessed the maximum SD value. Conversely, *CYP2* was the last in the SD value ranking, which meant it was probably the most stable among all candidate reference genes (Figure 1 and Table S2). In general, though Ct values can be parameters to make evaluations of the expression level and the stability of candidate reference genes, more systematical data analyses are still in needed to assess the reliability of all candidate genes under different environmental treatments.

### 3.3. The Analysis of Expression Stability of Candidate Reference Genes

For a more in-depth analysis of the qRT-PCR results of all candidates under different stresses, three kinds of software (geNorm, NormFinder, and BestKeeper) were used and all raw Ct values were pretreated and classified into a compatible form before being used.

#### 3.3.1. geNorm Analysis

**Figure 2:**
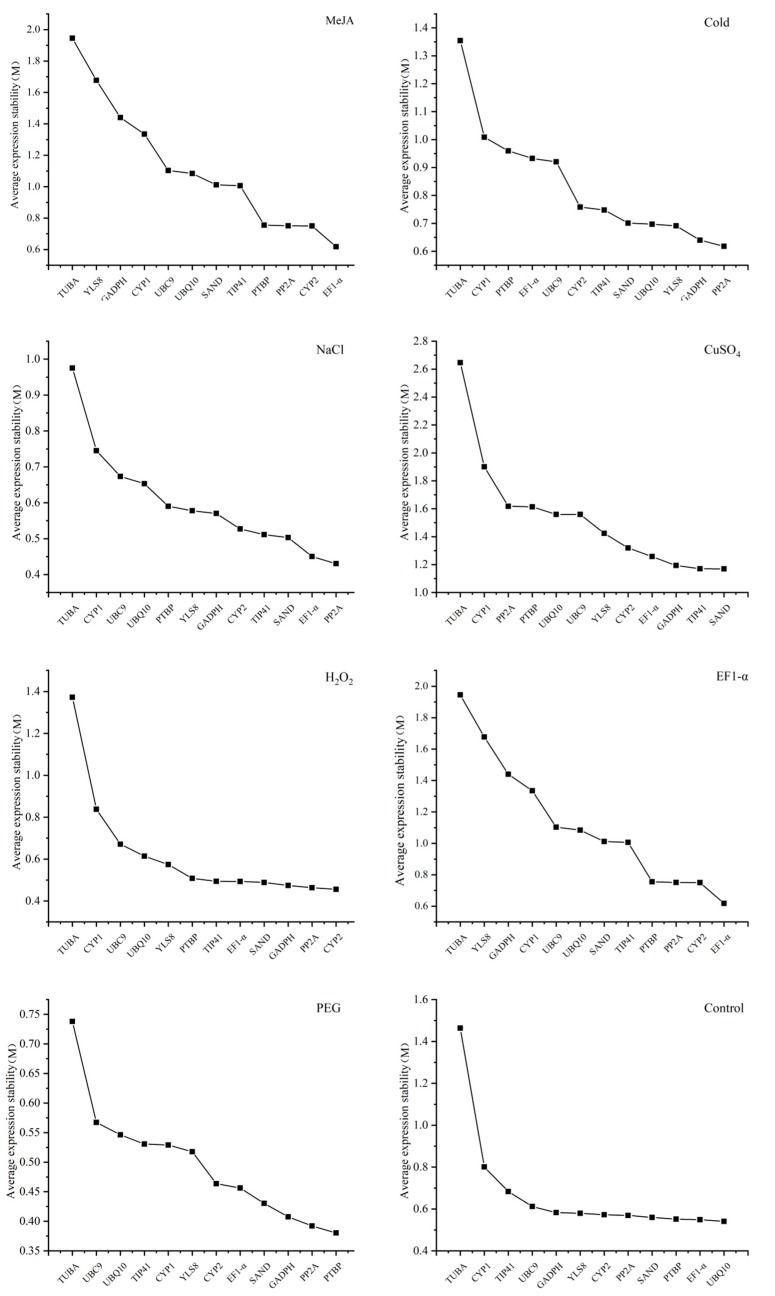
The rankings of the average expression stability (M) of 12 candidate reference genes in *C. yanhusuo* under 8 different treatments calculated by geNorm. The expression stability is assessed by the expression stability value (M).

According to geNorm analysis, *TUBA* showed the highest values under all pressure treatments, which demonstrated that *TUBA* had the worst stability. *PP2A* was the most stable candidate gene under salt and low-temperature treatment, as it showed the lowest M value. In addition, *PP2A* was the second most stable result under both drought and oxidative treatments, whereas it was unstable under MeJA, UV, and in the control group (Figure 2). According to Figure 2, there was no obvious M value change among the lowest three results in the CuSO_4_, H_2_O_2_, cold, and control groups. Moreover, the M values of *SAND*, *TIP41*, and *GAPDH* in CuSO_4_ were just the same, which meant that there were no big stability differences about the stability of *SAND*, *TIP41*, and *GAPDH* in the CuSO_4_ group. This phenomenon could also be found in *GAPDH*, *PP2A*, and *CYP2* in the H_2_O_2_ group, and *PTBP*, *EF1-α*, and *UBQ10* in the control group (Figure 2). 

**Figure 3:**
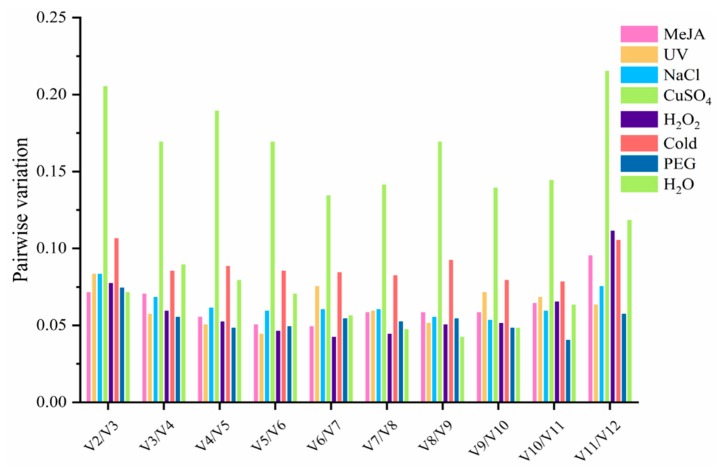
The pairwise variation values of all candidates calculated by geNorm. Different colors represent different ways of treatments. The lower the value, the better the stability of the combination. The cut-off value for assessing the number of candidate reference genes needed for qRT-PCR normalization is 0.15.

In addition to the function of assessing the stability of candidate gene expression, the other function of geNorm is to evaluate the optimal number of reference genes required for precise normalization. The geNorm algorithm uses pairwise variation (V_n_/V_n+1_) as a parameter, and 0.15 is usually regarded as the threshold value for normalization. A value lower than 0.15 represents that there will be no huge impact on normalization, even when one more reference gene is added, whereas a value more than 0.15 indicates that there will be a huge influence [16]. In this research, the V_n_/V_n+1_ could be obtained from geNorm (Figure 3 and Table S3) and the values of the four groups were all lower than 0.15. The V_2_/V_3_ values of all groups exposed to multifarious pressures fell below 0.15, except CuSO_4_, which indicated that one more reference gene added would not provide further improvement for the data normalization. For accurate normalization, metal treatment required at least six reference genes. Furthermore, the pairwise variation value of metal treatment is the only one that exceeded 0.15, and the pairwise variation values under other treatments were significantly less than 0.15 (Figure 3). However, pairwise variation is not a strict parameter and 0.15 is not a precise standard for apprising the number of reference genes needed for precise normalization [16]. One more reference gene is still recommended for precise data normalization in qRT-PCR analysis.

#### 3.3.2. NormFinder Analysis

The data calculated by NormFinder is listed in Table 2. In all samples under different environmental conditions calculated by NormFinder, *UBQ10* showed the most stability under MeJA analysis as it had the lowest value. There were four groups of M values (MeJA, NaCl, H_2_O_2_, cold) lower than 0.1 and they were *UBQ10* (0.024), *PP2A* (0.042), *CYP2* (0.039), and *PP2A* (0.097), respectively. In the other four groups (UV, CuSO_4_, PEG, and control), *GAPDH* (0.147), *TIP41* (0.185), *PTBP* (0.100), and *UBQ10* (0.168) were the most reliable reference gene candidates. *TUBA* had the lowest M value in all groups, which indicated that *TUBA* had the lowest stability among all candidate genes. In addition, *UBC9* also showed a low M value in most of the treatments. These results also aligned with the results of the geNorm analysis. The NormFinder analysis had very similar results with the geNorm analysis, especially in the following genes: *GAPDH*, *PP2A*, *TIP41*, *CYP2*, and *PTBP*.

#### 3.3.3. BestKeeper Analysis

Ct values can be directly processed by BestKeeper analysis instead of transferring to relative expression levels [34]. The SD and CV were the parameters that evaluated the stability and expression level of candidate genes, as shown in Table 3. The values of CV ± SD can assess the stability of candidate genes, and lower CV ± SD values represent more stable genes. For another measurement, genes would be considered unstable if the SD value was more than 1.00. *YLS8* was the most stable candidate under the UV, CuSO_4_, and cold treatments with the lowest CV ± SD value at 1.50 ± 0.32, 2.25 ± 0.43, and 0.86 ± 0.19, respectively. In NaCl and H_2_O_2_ conditions, *SAND* was the most stable gene with CV ± SD values of 1.09 ± 0.23 and 0.85 ± 0.18. In the MeJA, PEG, and control groups, *UBC9*, *PP2A*, and *TIP41* showed the most stability, with CV ± SD values of 1.77 ± 0.38, 1.26 ± 0.24, and 6.15 ± 1.40, respectively. However, *UBC9* showed high instability, as it showed low stability in the NaCl, H_2_O_2_, and PEG groups. In addition, in most treatments, *TUBA* and *CYP1* both exhibited lower stability than the other candidate genes, which is consistent with the geNorm and NormFinder analyses.

### 3.4. Comprehensive Analysis and Validation of Reference Genes

**Figure 4:**
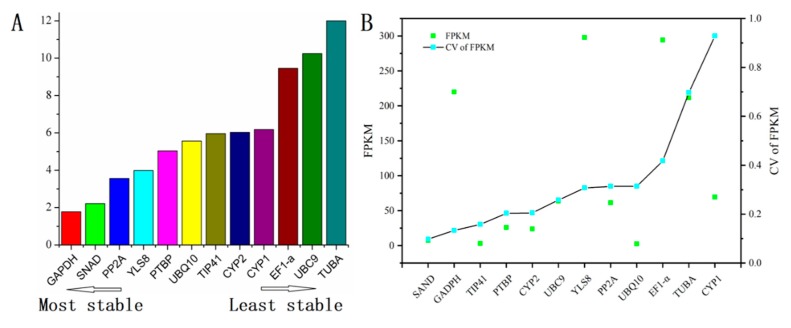
The order of stability of candidate genes by RefFinder and CV of FPKM (fragments per kilobase of exon model per million mapped fragments) in RNA-seq. (**A**) The order of stability of candidate genes by RefFinder (**B**) The order of stability of candidate genes by CV of FPKM. Lower CV values indicate more stable gene expression.

**Figure 5:**
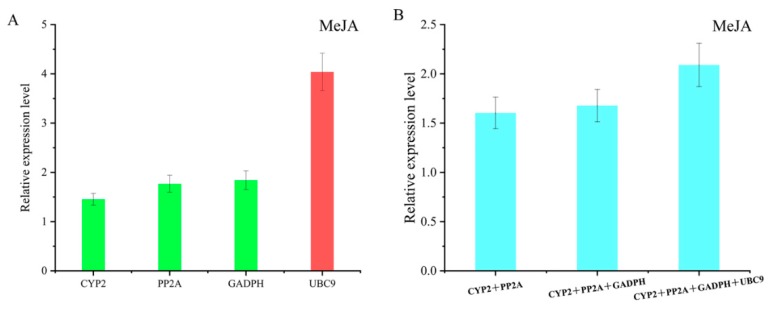
Relative expression level of *ACO* normalized by identified reference genes under methyl jasmonate (MeJA) treatment. (**A**) Expression level was normalized by the most stable reference genes and least stable gene (**B**) Expression level normalized by combinations. Data were exhibited as means ± SEM (*n* = 3).

To identify the most reliable gene under various environmental conditions, a comprehensive ranking platform RefFinder was performed and the results are listed in Figure 4A. As it is indicated, *GAPDH*, *SNAD*, and *PP2A* were the most stable candidate genes in most situations. However, *UBC9*, *TUBA* and *EF1-α* are considered to be the least stable reference genes. To validate these results, the CV of FPKM (fragments per kilobase of exon model per million mapped fragments) of all selected genes were employed and the results are listed in Figure 4B. In addition, the CV value of FPKM can represent the variability of gene expression. According to Figure 4B, the CV values of *SAND*, *GAPDH*, and *TIP41* were lower than that of other genes, indicating a more stable expression. In order to further verify the ranking results of the candidate genes under three algorithms, the *ACO* gene was used as a standard to verify the stability of candidate genes. *ACO* is a functional enzyme involved in the biosynthesis of ethylene in plants [36]. Ethylene is a kind of plant regulator that can contribute to tolerance under abiotic stresses, which include cold and drought treatments [37,38]. Therefore, *ACO* can be considered as a constantly expressed gene to validate the candidates we selected. According to Figure 5A, the expression level of *ACO* was slightly upregulated by using *CYP2*, *PP2A*, and *GAPDH* as reference genes under MeJA treatment. Nevertheless, a significant difference could be observed when *UBC9* was used to normalize the expression level of *ACO*. *UBC9* was selected as the unstable gene to perform the validation experiment, as it showed relative instability under all treatments and analysis methods while some other genes, like *TUBA* and *CYP1*, were stable under some treatments or analytic methods, even though they may be extremely unstable in most cases. To further validate the selected genes, combinations among the most stable candidate genes were imposed to analyze the expression of *ACO*, and similar results were exhibited. However, when unstable *UBC9* was added to the combination, the results did not show an obvious difference (Figure 5B). According to the results of the geNorm, the optimal number of genes required to combine to achieve precise normalization was obtained. This suggested that *UBC9* was not a stable reference gene when used alone, but it could be advised in combination with other reference genes to ensure normalization.

## 4. Discussion

Quantitative RT-PCR is a commonly and widely used technology with high specificity and sensitivity. It is used for high-throughput analysis of transcript levels and it has a repeated quantitative dynamic range. It plays a crucial part in quantifying the abundance of target genes and increasing the quantitative accuracy of target genes in different species [39,40]. However, erroneous conclusions of targeted gene normalization can be obtained if an inappropriate reference gene is selected [41,42]. Thus, selecting a suitable reference gene is very crucial for target genes’ normalization and data analysis [43]. Validating reference genes under certain environmental treatments and in different species is necessary [44,45]. Though the extract of *C. yanhusuo* has been used in promoting blood circulation, inhibiting cancer cell proliferation, and as an analgesic agent, no reliable reference genes have been selected in this species [2,16,46]. In order to determine the most reliable reference gene, 12 candidate genes were chosen based on the TAIR dataset and previous research. The Ct values of *C. yanhusuo* samples pretreated under different abiotic environments were obtained from qRT-PCR and processed by geNorm, NormFinder, and BestKeeper. According to the results of this study, the accuracy of the experimental results can only be guaranteed when reference genes are specifically selected according to different environments. Despite the same raw Ct data being applied to geNorm, NormFinder, and BestKeeper, the rankings of candidate genes were different. NormFinder and geNorm had similar analysis results, such as for *PP2A*, *GAPDH*, and *YLS8* in the cold group, and *PTBP*, *PP2A*, and *GAPDH* in the PEG group. In addition, in the UV, NaCl, CuSO_4_, and control groups, both NormFinder and geNorm had similar analysis results in the first three rankings. Nevertheless, in both the hormone treatment and metal treatment groups, NormFinder and BestKeeper showed different results; *UBQ10* and *TIP41* in NormFinder while *EF1-α* and *SAND* in geNorm (Figure 2 and Table 2). The BestKeeper analysis, however, had a very different result from that of NormFinder and geNorm. For example, in the low-temperature treatment group, NormFinder selected *PP2A* as the best candidate while *YLS8* was selected by the BestKeeper analysis. Interestingly, the reference genes selected by BestKeeper under different groups were different from those of NormFinder and geNorm (Figure 2, Table 2 and Table 3). A possible reason for this is that NormFinder and geNorm have similar processing methods and algorithms for raw data while BestKeeper uses CV ± SD to rank the stability.

The major and general functions of reference genes are to take part in the cell expression process and cellular structural components. Genes like *18S rRNA* and *EF1-α* are usually identified as the constantly expressed gene under different pressure conditions [25,47]. However, it has been proven by previous research that reference genes will not always be stalely expressed under certain circumstances or in various species [48,49]. In this experiment, for example, *PP2A* has been proven as the most stable gene under NaCl and cold treatment while it was unstable under UV treatment, ranking in the middle among all candidate genes. When compared with previous research, similar results were obtained in this study, showing *PP2A* to be stable under salt treatment and unstable under drought treatment [8]. Beyond that, though *GAPDH* worked very well under the UV and low-temperature treatment, the same results could not be drawn from MeJA and the control group. Similar results can also be found from the drought group in *Salicornia europaea* [50]. *UBQ10* was the most stable gene in the control group, but it is unstable in lettuce under abscisic acid treatment [51]. *GAPDH* was demonstrated as the most stable gene by the simulation of UV in *C. yanhusuo*, but it was reported to be unstable by geNorm analysis under salt treatment in maize [52]. The results of this study and previous research remind us that full consideration of environmental conditions and species should be taken into account when appropriate candidate reference genes are chosen [26]. To determine reliable reference genes under a set of experimental pressures, systematic data analysis is necessary. The most stable reference gene indicated by using each software under certain experimental conditions could be an appropriate choice. However, genes ranked in the second and third position may also have similar stability characteristics, which indicates that not only one internal reference gene could be used for accurate standardization of qRT-PCR in a particular state. For instance, the oxidative, metal, and control groups showed a flat and steady end curve, which means the oxidative, metal, and control groups each have three stable reference genes (Figure 2). In addition, studies have shown that the accurate normalization of target genes may not be guaranteed by only a single reference gene [53,54]. Therefore, in order to solve this problem, it is recommended to use 0.15 as an ideal threshold to determine the number of reference genes required for normalization under various environmental stress conditions [27]. The pairwise variation results are shown in Figure 3, where the V_2_/V_3_ values of all samples under various treatments, except metal, are below 0.15, which indicates that two reference genes were enough, and an additional candidate gene was not necessary for accurate quantification. Nevertheless, 0.15 is a reference parameter obtained from previous studies and is a theoretical threshold instead of an absolute value [39].

Considering the most stable genes were different in different situations, a comprehensive ranking platform, RefFinder, was employed to identify the most reliable gene under various environmental conditions. The results display a high consistency to that of the NormFinder, geNorm, BestKeeper, and Ct values (Figure 1, Figure 2, and Figure 4). In addition, the CV values of FPKM of all candidate genes were compared with different groups in RNA-seq data [12]. Interestingly, it also has consistent results with RefFinder, in that *GAPDH* and *SAND* are the two most stable reference genes in the two methods. Furthermore, to further validate the selected reference gene, the *ACO* gene was selected as a standard. According to similar results from previous studies, *ACO* is very stable and can be slightly regulated [37,38]. Our result is consistent with a previous report using the most stable gene as the reference gene. Using the least stable reference gene would lead to a significantly different result, in which the expression of *ACO* is very high. It shows that the stability and abundance of the reference gene not only affects the normalization results but also indicates that the stability of the reference gene needs to be evaluated before being used for a set of samples.

## 5. Conclusions

In conclusion, six candidate genes were obtained by comparing them with previous studies, and the remaining six candidate reference genes were selected according to the TAIR database. All candidate reference genes were pretreated under eight different treatments to select the most reliable one. The experimental results showed that the candidate reference genes had different stabilities under different treatments, and different algorithms also showed slightly different results. Generally speaking, in most situations, *GAPDH*, *SNAD*, and *PP2A* were the most stable candidate genes that could be used for normalization. In addition, *UBC9*, *TUBA*, and *EF1-α* can be considered as the least stable reference genes among the 12 candidates. The optimal number of reference genes needed for normalization was also evaluated by geNorm and the results indicated that, in most treatments, one reference gene is enough for normalization. Beyond that, the evaluation was performed by comparing the analyses with the RNA-seq-based expression profile to verify the experimental results, and *ACO* was also used to verify the reliability of the rankings of all candidate reference genes. In general, the result of this research can benefit studies that require accurate quantification of gene expression in *C. yanhusuo*, and it can also provide guidelines for researchers who aim to seek out the best reference genes in other plants.

## Figures and Tables

**Table 1:** Candidate genes and primer pairs used for qRT-PCR in *Corydalis yanhusuo*.

Gene symbol	Description	Gene ID	*Arabidopsis* Homolog Locus	Primer Sequence Forward/Reverse (5′–3′)	Length (bp)	PCR Efficiency	R^2^
*CYP2*	Cyclophilin 2	XP_008340167.1	AT4G33060	F: TGGTGCATCACTTGCTATGG R: GTTGTTTGGCTCCACCACTA	164	1.848	0.960
*EF1-α*	Elongation factor 1-α	XP_018856763.1	AT1G07920	F: CTGCCCCTTCAGGATGTTTA R: GCCTCGTGATGCATTTCAAC	152	1.803	0.881
*PP2A*	Serine/threonine-protein phosphatase PP2A	OVA18136.1	ATG59830	F: TCCCCATCTATCGAGACCCT R: GTCCTGGCCAAATGTGTATC	124	1.828	0.960
*PTBP*	Polypyrimidine tract-binding protein	OVA06588.1	AT3G01150	F: AGCCAGGGCAGTTGCTTATC R: CCAGGACAGTGCATCTTTCG	134	1.799	0.841
*SAND*	SAND family protein	XP_010260994.1	AT2G28390	F: AGATGGTGGCCTACGTGTTG R: GCCAATGTCAGCTTCCTTGA	130	1.858	1.000
*TIP41*	TIP41-like protein	XP_010260049.1	AT4G34270	F: GTCATGCCGAGTTGTTGGTT R: AAATGTGGCTTCTCTCCAGC	153	1.796	0.841
*UBC9*	Ubiquitin-conjugating enzyme 9	OVA15929.1	AT4G27960	F: TGGCAAGCAACAATTATGGG R: GCAGATGCTTCCATTGCTGT	159	1.788	0.841
*UBQ10*	Ubiquitin-conjugating enzyme 10	XP_010261482.1	AT4G05320	F: CATCCAGAAGGAGTCTACCC R: AGCTTTCACGTTATCAATCG	140	1.815	0.960
*CYP1*	Cyclophilin 1	AAN31845.1	AT2G16600	F: TTCCAAAGTTTCAGAGTCCC R: CATGTGCTTGGGATTCAATC	136	1.747	0.907
*TUBA*	Tubulin beta	OVA16215.1	AT5G12250	F: TTGACCTCTGCTTAGACCGC R: GTGAACCCAATCCAGAACCA	111	1.598	0.676
*YLS8*	Mitosis protein	KJB77370.1	AT5G08290	F: ACTTGTCGTAATTCGGTTCG R: CAACAAGGTAGATCACCGCA	124	1.765	0.815
*GAPDH*	Glyceraldehyde-3-phosphate dehydrogenase	XP_010941981.2	AT1G42970	F: CAAGGTCATCAACGACAGGT R: TGCTGCTGGGAATGATGTTG	149	1.860	1.000

**Table 2:** The stability of the candidate genes expression calculated by NormFinder software.

Rank	MeJA	UV	NaCl	CuSO_4_	H_2_O_2_	Cold	PEG	Control
1	*UBQ10* 0.024	*GAPDH* 0.147	*PP2A* 0.042	*TIP41* 0.185	*CYP2* 0.039	*PP2A* 0.097	*PTBP* 0.100	*UBQ10* 0.168
2	*PTBP* 0.054	*EF1-α* 0.173	*EF1-α* 0.063	*SAND* 0.187	*GAPDH* 0.055	*GAPDH* 0.134	*PP2A* 0.114	*SAND* 0.183
3	*GAPDH* 0.101	*UBC9* 0.199	*CYP2* 0.200	*GAPDH* 0.207	*PP2A* 0.076	*YLS8* 0.182	*GAPDH* 0.130	*PP2A* 0.190
4	*EF1-α* 0.156	*TIP41* 0.207	*SAND* 0.210	*EF1-α* 0.382	*EF1-α* 0.140	*SAND* 0.267	*SAND* 0.166	*PTBP* 0.205
5	*PP2A* 0.230	*UBQ10* 0.220	*TIP41* 0.217	*CYP2* 0.531	*SAND* 0.183	*UBQ10* 0.277	*CYP2* 0.196	*YLS8* 0.216
6	*UBC9* 0.240	*CYP2* 0.278	*GAPDH* 0.246	*YLS8* 0.555	*TIP41* 0.193	*TIP41* 0.293	*EF1-α* 0.229	*CYP2* 0.230
7	*TIP41* 0.251	*SAND* 0.280	*YLS8* 0.267	*UBC9* 0.781	*PTBP* 0.218	*CYP2* 0.346	*YLS8* 0.264	*EF1-α* 0.236
8	*CYP1* 0.306	*PP2A* 0.317	*PTBP* 0.308	*UBQ10* 0.798	*YLS8* 0.276	*UBC9* 0.515	*CYP1* 0.274	*GAPDH* 0.270
9	*SAND* 0.339	*PTBP* 0.351	*UBC9* 0.371	*PTBP* 0.961	*UBQ10* 0.285	*PTBP* 0.557	*UBQ10* 0.287	*UBC9* 0.313
10	*CYP2* 0.403	*YLS8* 0.406	*UBQ10* 0.372	*PP2A* 0.969	*UBC9* 0.339	*EF1-α* 0.559	*TIP41* 0.307	*TIP41* 0.373
11	*YLS8* 0.502	*CYP1* 0.503	*CYP1* 0.430	*CYP1* 1.213	*CYP1* 0.526	*CYP1* 0.605	*UBC9* 0.330	*CYP1* 0.420
12	*TUBA* 0.797	*TUBA* 0.527	*TUBA* 0.626	*TUBA* 1.782	*TUBA* 0.925	*TUBA* 0.871	*TUBA* 0.476	*TUBA* 0.985

**Table 3:** The stability of the candidate genes expression calculated by BestKeeper software.

Rank	MeJA	UV	NaCl	CuSO_4_	H_2_O_2_	Cold	PEG	Control (H_2_O)
1	*UBC9*	*YLS8*	*SAND*	*YLS8*	*SAND*	*YLS8*	*PP2A*	*TIP41*
CV ± SD	1.77 ± 0.38	1.50 ± 0.32	1.09 ± 0.23	2.25 ± 0.43	0.85 ± 0.18	0.86 ± 0.19	1.26 ± 0.24	6.15 ± 1.40
2	*CYP1*	*UBC9*	*YLS8*	*GAPDH*	*YLS8*	*TIP41*	*TIP41*	*TUBA*
CV ± SD	1.95 ± 0.35	1.50 ± 0.36	1.10 ± 0.22	2.28 ± 0.40	0.94 ± 0.19	1.29 ± 0.31	1.40 ± 0.33	6.64 ± 1.88
3	*PTBP*	*CYP1*	*PP2A*	*SAND*	*EF1-α*	*UBC9*	*PTBP*	*EF1-α*
CV ± SD	2.08 ± 0.41	1.55 ± 0.28	1.47 ± 0.26	2.82 ± 0.58	1.32 ± 0.30	1.96 ± 0.48	1.44 ± 0.30	7.06 ± 1.67
4	*YLS8*	*UBQ10*	*EF1-α*	*TIP41*	*TIP41*	*GAPDH*	*SAND*	*UBC9*
CV ± SD	2.12 ± 0.43	2.22 ± 0.42	1.48 ± 0.32	2.82 ± 0.59	1.50 ± 0.32	2.14 ± 0.43	1.50 ± 0.32	7.56 ± 1.68
5	*TIP41*	*TIP41*	*TIP41*	*UBC9*	*PTBP*	*PP2A*	*CYP1*	*GAPDH*
CV ± SD	2.21 ± 0.48	2.31 ± 0.5	1.61 ± 0.35	3.80 ± 0.75	1.61 ± 0.31	2.17 ± 0.46	1.65 ± 0.28	7.97 ± 1.63
6	*GAPDH*	*CYP2*	*UBQ10*	*EF1-α*	*CYP2*	*PTBP*	*YLS8*	*PTBP*
CV ± SD	2.24 ± 0.41	2.32 ± 0.41	1.74 ± 0.33	3.94 ± 0.83	1.76 ± 0.32	2.28 ± 0.52	1.84 ± 0.38	8.20 ± 1.70
7	*EF1-α*	*TUBA*	*PTBP*	*UBQ10*	*PP2A*	*TUBA*	*EF1-α*	*SAND*
CV ± SD	2.24 ± 0.50	2.42 ± 0.54	2.38 ± 0.44	5.10 ± 0.92	2.05 ± 0.36	2.74 ± 0.74	2.11 ± 0.44	8.27 ± 1.86
8	*UBQ10*	*EF1-α*	*CYP1*	*PTBP*	*CYP1*	*SAND*	*UBQ10*	*UBQ10*
CV ± SD	2.43 ± 0.47	2.73 ± 0.54	2.43 ± 0.40	5.72 ± 1.10	2.09 ± 0.36	2.75 ± 0.63	2.13 ± 0.38	8.29 ± 1.68
9	*SAND*	*GAPDH*	*GAPDH*	*TUBA*	*GAPDH*	*CYP2*	*TUBA*	*CYP1*
CV ± SD	3.18 ± 0.66	2.82 ± 0.52	2.60 ± 0.47	5.89 ± 1.49	2.37 ± 0.42	2.91 ± 0.62	2.24 ± 0.62	8.67 ± 1.63
10	*PP2A*	*SAND*	*CYP2*	*CYP1*	*UBQ10*	*UBQ10*	*GAPDH*	*YLS8*
CV ± SD	3.40 ± 0.60	3.13 ± 0.69	2.66 ± 0.46	6.00 ± 1.02	3.35 ± 0.61	3.16 ± 0.65	2.28 ± 0.42	9.21 ± 2.01
11	*CYP2*	*PTBP*	*TUBA*	*CYP2*	*UBC9*	*EF1-α*	*UBC9*	*PP2A*
CV ± SD	3.50 ± 0.61	3.42 ± 0.70	2.68 ± 0.73	6.00 ± 1.06	3.36 ± 0.70	4.15 ± 0.85	2.32 ± 0.47	9.90 ± 1.96
12	*TUBA*	*PP2A*	*UBC9*	*PP2A*	*TUBA*	*CYP1*	*CYP2*	*CYP2*
CV ± SD	4.23 ± 1.02	3.59 ± 0.66	3.18 ± 0.66	6.93 ± 1.24	4.51 ± 1.23	4.49 ± 0.80	2.66 ± 0.49	10.26 ± 2.0

CV: the coefficient of variance expressed as a percentage on the CP level; SD: the standard deviation of the CP.

## Supplementary Materials

The following are available online at https://www.mdpi.com/2073-4425/11/2/130/s1, Figure S1: The melting cures of 12 candidate reference genes, Table S1: The details of 12 selected candidate reference genes, Table S2: The raw Ct values of 12 candidate reference genes with three biological and technical replicates, Table S3: The Vn/Vn+1 information obtained from geNorm.
