# Supplementary material for: Identification and Selection of Reference Genes for Quantitative Transcript Analysis in Corydalis yanhusuo
Source: Genes (Basel). 2020 Jan 27;11(2):130. doi: 10.3390/genes11020130 (PMC7074024; doi:10.3390/genes11020130)
Supplement: Supplementary file 1 [file genes-11-00130-s001.zip › Supplemental File.docx]

**Supplementary Materials**

**Identification and Selection of Reference Genes for Quantitative Transcript Analysis in *Corydalis yanhusuo***

**Zhenzhen Bao ^1†^, Kaidi Zhang ^2†^, Hanfeng Lin ^2^, Changjian Li ^3^, Xiurong Zhao ^2^, Jie Wu^1*^ and Sihui Nian^4*^**

**Supplementary figures and tables**


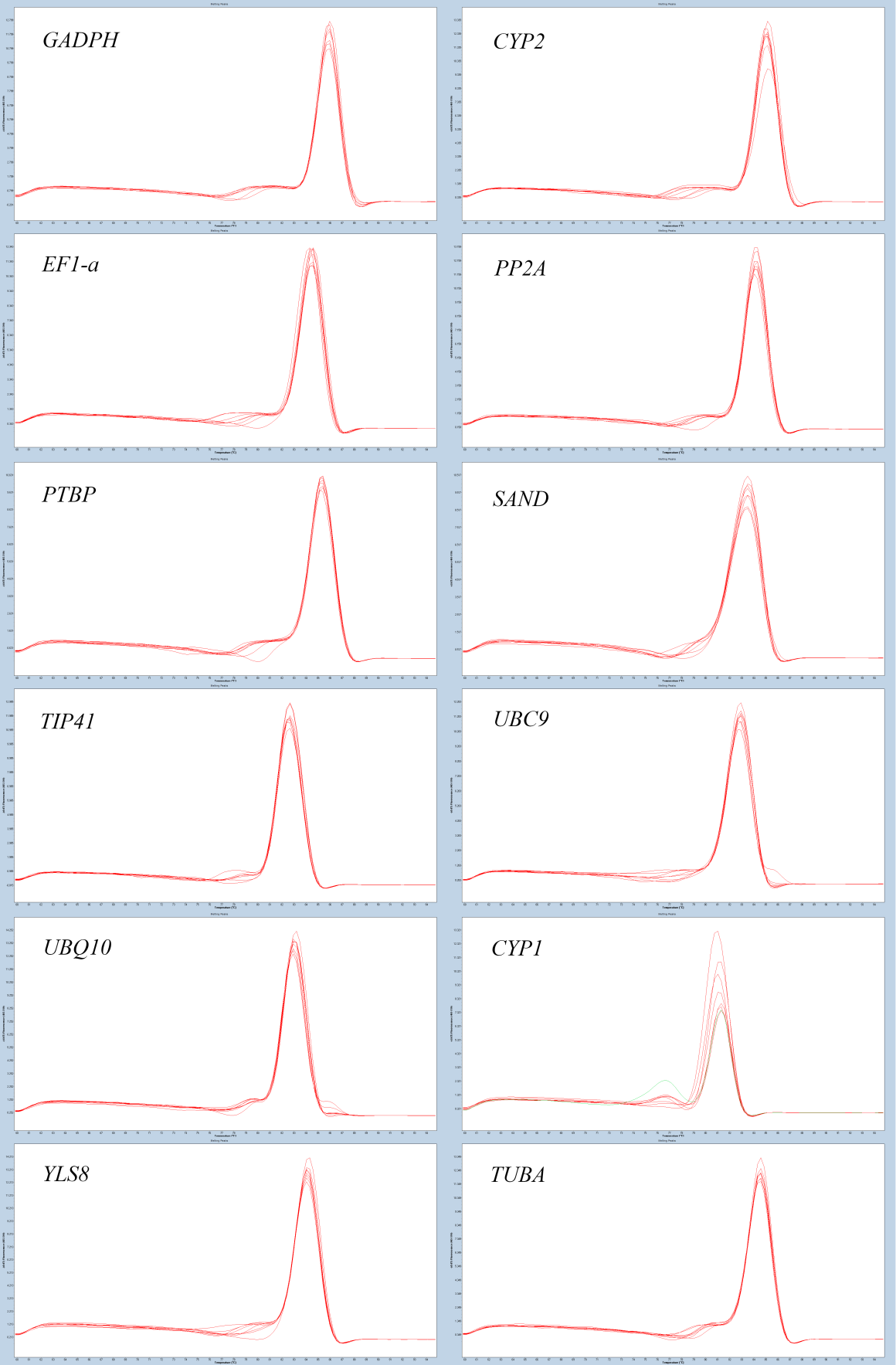


**Figure S1.** The melting curves of 12 candidate reference genes. X-axis shows temperature (℃), Y-axis shows fluorescence (465-510). The melting temperature for *GADPH, CYP2, EF1-α, PP2A, PTBP, SAND, TIP41, UBC9, UBQ10, CYP1, YLS8, TUBA* are 86, 85, 84, 84, 85, 83, 83, 83, 83, 81, 84, 84, respectively.

**Table S1.** The details of 12 selected candidate reference genes.

See the supported excel file.

**Table S2.** The raw Ct value of 12 candidate reference genes with three biological and technical replicates.

| Biological replicates | *CYP2* | *EF1-α* | *PP2A* | *SAND* | *PTBP* | *TIP41* | *UBC9* | *UBQ10* | *CYP1* | *TUBA* | *YLS8* | *GADPH* |
| --- | --- | --- | --- | --- | --- | --- | --- | --- | --- | --- | --- | --- |
| MeJA (1) | 17.63 | 21.88 | 17.31 | 20.88 | 19.44 | 21.72 | 21.26 | 18.94 | 17.37 | 24.66 | 19.56 | 18.39 |
|  | 15.96 | 21.87 | 16.69 | 19.42 | 18.72 | 21.43 | 21.15 | 18.58 | 17.86 | 23.03 | 20.66 | 17.48 |
|  | 17.57 | 22.13 | 17.72 | 20.96 | 19.63 | 21.42 | 20.95 | 19.42 | 18.21 | 24.87 | 20.59 | 18.33 |
| MeJA (2) | 17.74 | 23.69 | 19.55 | 22.47 | 20.88 | 23.35 | 22.78 | 20.57 | 18.75 | 25.49 | 20.96 | 19.37 |
|  | 17.81 | 22.83 | 17.90 | 21.54 | 19.66 | 22.00 | 21.71 | 19.50 | 18.18 | 24.64 | 21.62 | 18.76 |
|  | 18.03 | 22.74 | 18.20 | 21.55 | 19.71 | 22.48 | 21.34 | 19.64 | 17.69 | 21.65 | 20.64 | 18.79 |
| MeJA (2) | 17.63 | 21.88 | 17.31 | 20.88 | 19.44 | 21.72 | 21.26 | 18.94 | 17.37 | 24.66 | 19.56 | 18.39 |
|  | 15.96 | 21.87 | 16.69 | 19.42 | 18.72 | 21.43 | 21.15 | 18.58 | 17.86 | 23.03 | 20.66 | 17.48 |
|  | 17.57 | 22.13 | 17.72 | 20.96 | 19.63 | 21.42 | 20.95 | 19.42 | 18.21 | 24.87 | 20.59 | 18.33 |
| UV (1) | 17.68 | 19.69 | 18.56 | 21.91 | 20.81 | 23.59 | 24.41 | 18.51 | 17.87 | 21.99 | 21.28 | 18.41 |
|  | 16.89 | 18.73 | 16.95 | 20.75 | 18.90 | 22.06 | 23.6 | 18.34 | 18.49 | 22.56 | 21.58 | 17.14 |
|  | 17.46 | 20.20 | 18.49 | 22.36 | 20.79 | 23.27 | 23.84 | 18.81 | 18.72 | 21.55 | 21.32 | 18.40 |
| UV (2) | 17.77 | 20.77 | 19.85 | 23.53 | 21.70 | 24.16 | 24.69 | 19.81 | 18.41 | 23.88 | 22.63 | 19.44 |
|  | 18.53 | 20.30 | 18.82 | 22.70 | 20.75 | 23.50 | 24.21 | 18.69 | 17.97 | 22.82 | 21.75 | 18.78 |
|  | 18.35 | 20.06 | 19.29 | 22.71 | 20.82 | 23.92 | 24.54 | 19.76 | 18.43 | 22.23 | 20.96 | 18.59 |
| UV (3) | 17.68 | 19.69 | 18.56 | 21.91 | 20.81 | 23.59 | 24.41 | 18.51 | 17.87 | 21.99 | 21.28 | 18.41 |
|  | 16.89 | 18.73 | 16.95 | 20.75 | 18.9 | 22.06 | 23.60 | 18.34 | 18.49 | 22.56 | 21.58 | 17.14 |
|  | 17.46 | 20.20 | 18.49 | 22.36 | 20.79 | 23.27 | 23.84 | 18.81 | 18.72 | 21.55 | 21.32 | 18.40 |
| NaCl (1) | 17.62 | 21.67 | 17.60 | 20.73 | 18.99 | 21.99 | 21.26 | 19.20 | 15.75 | 26.82 | 19.90 | 17.94 |
|  | 16.63 | 20.87 | 16.91 | 20.21 | 17.61 | 20.94 | 19.76 | 18.67 | 16.71 | 25.89 | 20.26 | 17.68 |
|  | 16.79 | 21.70 | 17.57 | 20.85 | 18.91 | 21.57 | 20.48 | 19.21 | 16.42 | 26.83 | 20.34 | 17.98 |
| NaCl (2) | 17.77 | 22.22 | 17.80 | 20.51 | 18.68 | 21.75 | 21.79 | 18.73 | 17.44 | 29.90 | 20.78 | 19.38 |
|  | 16.76 | 21.75 | 17.30 | 20.50 | 18.80 | 20.97 | 21.54 | 17.92 | 15.88 | 26.61 | 20.66 | 18.59 |
|  | 17.56 | 21.85 | 17.56 | 20.91 | 18.44 | 21.45 | 21.46 | 18.72 | 16.43 | 27.6 | 20.55 | 18.83 |
| NaCl (3) | 17.62 | 21.67 | 17.6 | 20.73 | 18.99 | 21.99 | 21.26 | 19.20 | 15.75 | 26.82 | 19.90 | 17.94 |
|  | 16.63 | 20.87 | 16.91 | 20.21 | 17.61 | 20.94 | 19.76 | 18.67 | 16.71 | 26.74 | 20.26 | 17.68 |
|  | 16.79 | 21.70 | 17.57 | 20.85 | 18.91 | 21.57 | 20.48 | 19.21 | 16.42 | 26.83 | 20.34 | 17.98 |
| CuSO_4_ (1) | 16.81 | 20.92 | 17.22 | 20.44 | 18.75 | 20.95 | 20.57 | 17.80 | 16.00 | 24.91 | 18.98 | 17.57 |
|  | 16.60 | 19.71 | 16.91 | 19.69 | 17.75 | 19.85 | 19.70 | 17.54 | 16.70 | 26.16 | 19.64 | 16.86 |
|  | 17.15 | 21.58 | 16.73 | 19.88 | 18.55 | 21.00 | 19.47 | 18.22 | 16.00 | 25.55 | 19.42 | 17.75 |
| CuSO_4_ (2) | 19.64 | 20.79 | 21.54 | 21.57 | 22.80 | 20.52 | 17.46 | 15.45 | 21.32 | 19.42 | 18.56 | 17.89 |
|  | 19.33 | 22.45 | 18.72 | 20.88 | 19.34 | 21.88 | 20.94 | 19.69 | 16.78 | 27.4 | 18.69 | 18.93 |
|  | 18.74 | 22.78 | 18.97 | 21.42 | 20.31 | 22.61 | 20.66 | 20.23 | 17.19 | 27.78 | 20.29 | 17.84 |
| CuSO_4_ (3) | 16.81 | 20.92 | 17.22 | 20.44 | 18.75 | 20.95 | 20.57 | 17.80 | 16.00 | 24.91 | 18.98 | 17.57 |
|  | 16.60 | 19.71 | 16.91 | 19.69 | 17.75 | 19.85 | 19.70 | 17.54 | 16.70 | 26.16 | 19.64 | 16.86 |
|  | 17.15 | 21.58 | 16.73 | 19.88 | 18.55 | 21.00 | 19.47 | 18.22 | 16.00 | 25.55 | 19.42 | 17.75 |
| H_2_O_2_ (1) | 17.88 | 22.54 | 17.51 | 20.71 | 19.42 | 21.69 | 21.22 | 18.76 | 16.5 | 26.54 | 20.32 | 17.77 |
|  | 17.69 | 22.47 | 16.84 | 20.51 | 18.53 | 20.86 | 19.77 | 17.55 | 17.65 | 26.51 | 20.30 | 17.31 |
|  | 17.41 | 21.96 | 17.35 | 20.76 | 19.01 | 21.43 | 20.48 | 17.68 | 16.93 | 25.77 | 19.78 | 17.50 |
| H_2_O_2_ (2) | 18.79 | 23.25 | 18.45 | 21.14 | 19.47 | 21.86 | 22.41 | 19.56 | 16.87 | 30.86 | 20.32 | 18.66 |
|  | 17.88 | 22.42 | 17.67 | 20.83 | 18.98 | 21.76 | 21.48 | 17.84 | 16.98 | 28.52 | 20.42 | 18.44 |
|  | 18.42 | 23.11 | 17.96 | 21.20 | 19.42 | 21.99 | 21.41 | 18.50 | 17.46 | 27.72 | 20.31 | 18.33 |
| H_2_O_2_ (3) | 17.88 | 22.54 | 17.51 | 20.71 | 19.42 | 21.69 | 21.22 | 18.76 | 16.50 | 26.54 | 20.32 | 17.77 |
|  | 17.69 | 22.47 | 16.84 | 20.51 | 18.53 | 20.86 | 19.77 | 17.55 | 17.65 | 26.51 | 20.30 | 17.31 |
|  | 17.41 | 21.96 | 17.35 | 20.76 | 19.01 | 21.43 | 20.48 | 17.68 | 16.93 | 25.77 | 19.78 | 17.50 |
| Cold 1 (1) | 22.22 | 21.37 | 21.61 | 23.52 | 22.94 | 23.90 | 24.79 | 21.38 | 17.76 | 26.75 | 22.48 | 20.26 |
|  | 21.72 | 20.55 | 20.70 | 22.35 | 21.79 | 23.93 | 23.4 | 20.53 | 18.66 | 27.59 | 22.77 | 19.89 |
|  | 21.80 | 21.74 | 21.73 | 23.79 | 23.37 | 24.46 | 24.45 | 21.57 | 18.57 | 26.48 | 22.82 | 20.57 |
| Cold (2) | 20.66 | 18.97 | 21.16 | 22.37 | 23.8 | 24.64 | 25.4 | 20.38 | 16.28 | 29.67 | 22.51 | 19.68 |
|  | 20.26 | 19.54 | 20.44 | 22.08 | 23.00 | 23.72 | 24.33 | 19.77 | 16.66 | 26.8 | 22.08 | 19.41 |
|  | 20.61 | 19.51 | 20.71 | 22.73 | 23.59 | 24.46 | 24.85 | 19.54 | 16.68 | 26.39 | 22.46 | 18.88 |
| Cold (3) | 22.22 | 21.37 | 21.61 | 23.52 | 22.94 | 23.90 | 24.79 | 21.38 | 17.76 | 26.75 | 22.48 | 20.26 |
|  | 21.72 | 20.55 | 20.70 | 22.35 | 21.79 | 23.93 | 23.4 | 20.53 | 18.66 | 27.59 | 22.77 | 19.89 |
|  | 21.80 | 21.74 | 21.73 | 23.79 | 23.37 | 24.46 | 24.45 | 21.57 | 18.57 | 26.48 | 22.82 | 20.57 |
| PEG (1) | 18.93 | 21.68 | 19.28 | 21.37 | 21.44 | 23.72 | 21.45 | 18.56 | 16.90 | 27.95 | 20.98 | 19.36 |
|  | 18.43 | 20.40 | 18.52 | 20.82 | 20.52 | 22.56 | 19.85 | 18.17 | 17.28 | 28.68 | 21.17 | 18.34 |
|  | 18.55 | 21.25 | 18.87 | 21.43 | 20.84 | 23.41 | 20.33 | 17.31 | 16.61 | 28.10 | 20.60 | 18.70 |
| PEG (2) | 17.62 | 20.64 | 18.62 | 20.62 | 20.62 | 23.41 | 20.52 | 17.86 | 16.39 | 27.04 | 19.92 | 18.27 |
|  | 17.47 | 20.48 | 18.76 | 20.55 | 20.30 | 23.19 | 20.55 | 17.67 | 16.30 | 26.79 | 20.44 | 17.97 |
|  | 17.54 | 20.93 | 18.56 | 21.00 | 20.71 | 23.35 | 19.80 | 17.80 | 16.73 | 26.70 | 19.96 | 17.94 |
| PEG (3) | 18.93 | 21.68 | 19.28 | 21.37 | 21.44 | 23.72 | 21.45 | 18.56 | 16.90 | 27.95 | 20.98 | 19.36 |
|  | 18.43 | 20.40 | 18.52 | 20.82 | 20.52 | 22.56 | 19.85 | 18.17 | 17.28 | 28.68 | 21.17 | 18.34 |
|  | 18.55 | 21.25 | 18.87 | 21.43 | 20.84 | 23.41 | 20.33 | 17.31 | 16.61 | 28.10 | 20.60 | 18.70 |
| H_2_O (1) | 18.4 | 22.92 | 18.42 | 21.27 | 19.90 | 22.31 | 21.66 | 19.27 | 16.95 | 25.53 | 20.26 | 19.56 |
|  | 17.95 | 21.90 | 18.49 | 20.71 | 18.91 | 21.54 | 20.88 | 19.22 | 18.35 | 27.49 | 20.37 | 18.58 |
|  | 18.18 | 22.40 | 17.98 | 21.35 | 19.55 | 21.43 | 20.54 | 18.71 | 17.59 | 27.53 | 20.32 | 19.40 |
| H_2_O (2) | 22.68 | 26.16 | 22.96 | 25.57 | 23.68 | 24.86 | 24.53 | 22.83 | 21.46 | 33.51 | 24.80 | 22.74 |
|  | 22.51 | 25.98 | 22.18 | 24.46 | 22.70 | 24.39 | 24.39 | 22.29 | 20.80 | 29.86 | 24.56 | 22.59 |
|  | 22.98 | 26.36 | 22.96 | 25.87 | 23.46 | 25.5 | 25.53 | 23.45 | 21.66 | 29.85 | 25.16 | 23.18 |
| H_2_O (3) | 18.40 | 22.92 | 18.42 | 21.27 | 19.90 | 22.31 | 21.66 | 19.27 | 16.95 | 25.53 | 20.26 | 19.56 |
|  | 17.95 | 21.90 | 18.49 | 20.71 | 18.91 | 21.54 | 20.88 | 19.22 | 18.35 | 27.49 | 20.37 | 18.58 |
|  | 18.18 | 22.40 | 17.98 | 21.35 | 19.55 | 21.43 | 20.54 | 18.71 | 17.59 | 27.53 | 20.32 | 19.40 |

**Table S3.** The Vn/Vn+1 information obtained from geNorm.

| Rank | MeJA | UV | NaCl | CuSO_4_ | H_2_O_2_ | Cold | PEG | Control (H_2_O) |
| --- | --- | --- | --- | --- | --- | --- | --- | --- |
| 1 | *UBC9* | *YLS8* | *SAND* | *YLS8* | *SAND* | *YLS8* | *PP2A* | *TIP41* |
| CV ± SD | 1.77 ± 0.38 | 1.50 ± 0.32 | 1.09 ± 0.23 | 2.25 ± 0.43 | 0.85 ± 0.18 | 0.86 ± 0.19 | 1.26 ± 0.24 | 6.15 ± 1.40 |
| 2 | *CYP1* | *UBC9* | *YLS8* | *GADPH* | *YLS8* | *TIP41* | *TIP41* | *TUBA* |
| CV ± SD | 1.95 ± 0.35 | 1.50 ± 0.36 | 1.10 ± 0.22 | 2.28 ± 0.40 | 0.94 ± 0.19 | 1.29 ± 0.31 | 1.40 ± 0.33 | 6.64 ± 1.88 |
| 3 | *PTBP* | *CYP1* | *PP2A* | *SAND* | *EF1-α* | *UBC9* | *PTBP* | *EF1-α* |
| CV ± SD | 2.08 ± 0.41 | 1.55 ± 0.28 | 1.47 ± 0.26 | 2.82 ± 0.58 | 1.32 ± 0.30 | 1.96 ± 0.48 | 1.44 ± 0.30 | 7.06 ± 1.67 |
| 4 | *YLS8* | *UBQ10* | *EF1-α* | *TIP41* | *TIP41* | *GADPH* | *SAND* | *UBC9* |
| CV ± SD | 2.12 ± 0.43 | 2.22 ± 0.42 | 1.48 ± 0.32 | 2.82 ± 0.59 | 1.50 ± 0.32 | 2.14 ± 0.43 | 1.50 ± 0.32 | 7.56 ± 1.68 |
| 5 | *TIP41* | *TIP41* | *TIP41* | *UBC9* | *PTBP* | *PP2A* | *CYP1* | *GADPH* |
| CV ± SD | 2.21 ± 0.48 | 2.31 ± 0.5 | 1.61 ± 0.35 | 3.80 ± 0.75 | 1.61 ± 0.31 | 2.17 ± 0.46 | 1.65 ± 0.28 | 7.97 ± 1.63 |
| 6 | *GADPH* | *CYP2* | *UBQ10* | *EF1-α* | *CYP2* | *PTBP* | *YLS8* | *PTBP* |
| CV ± SD | 2.24 ± 0.41 | 2.32 ± 0.41 | 1.74 ± 0.33 | 3.94 ± 0.83 | 1.76 ± 0.32 | 2.28 ± 0.52 | 1.84 ± 0.38 | 8.20 ± 1.70 |
| 7 | *EF1-α* | *TUBA* | *PTBP* | *UBQ10* | *PP2A* | *TUBA* | *EF1-α* | *SAND* |
| CV ± SD | 2.24 ± 0.50 | 2.42 ± 0.54 | 2.38 ± 0.44 | 5.10 ± 0.92 | 2.05 ± 0.36 | 2.74 ± 0.74 | 2.11 ± 0.44 | 8.27 ± 1.86 |
| 8 | *UBQ10* | *EF1-α* | *CYP1* | *PTBP* | *CYP1* | *SAND* | *UBQ10* | *UBQ10* |
| CV ± SD | 2.43 ± 0.47 | 2.73 ± 0.54 | 2.43 ± 0.40 | 5.72 ± 1.10 | 2.09 ± 0.36 | 2.75 ± 0.63 | 2.13 ± 0.38 | 8.29 ± 1.68 |
| 9 | *SAND* | *GADPH* | *GADPH* | *TUBA* | *GADPH* | *CYP2* | *TUBA* | *CYP1* |
| CV ± SD | 3.18 ± 0.66 | 2.82 ± 0.52 | 2.60 ± 0.47 | 5.89 ± 1.49 | 2.37 ± 0.42 | 2.91 ± 0.62 | 2.24 ± 0.62 | 8.67 ± 1.63 |
| 10 | *PP2A* | *SAND* | *CYP2* | *CYP1* | *UBQ10* | *UBQ10* | *GADPH* | *YLS8* |
| CV ± SD | 3.40 ± 0.60 | 3.13 ± 0.69 | 2.66 ± 0.46 | 6.00 ± 1.02 | 3.35 ± 0.61 | 3.16 ± 0.65 | 2.28 ± 0.42 | 9.21 ± 2.01 |
| 11 | *CYP2* | *PTBP* | *TUBA* | *CYP2* | *UBC9* | *EF1-α* | *UBC9* | *PP2A* |
| CV ± SD | 3.50 ± 0.61 | 3.42 ± 0.70 | 2.68 ± 0.73 | 6.00 ± 1.06 | 3.36 ± 0.70 | 4.15 ± 0.85 | 2.32 ± 0.47 | 9.90 ± 1.96 |
| 12 | *TUBA* | *PP2A* | *UBC9* | *PP2A* | *TUBA* | *CYP1* | *CYP2* | *CYP2* |
| CV ± SD | 4.23 ± 1.02 | 3.59 ± 0.66 | 3.18 ± 0.66 | 6.93 ± 1.24 | 4.51 ± 1.23 | 4.49 ± 0.80 | 2.66 ± 0.49 | 10.26 ± 2.0 |
